# Automated generation of personalized trajectories of aging phenotypes with DyViA-GAN

**DOI:** 10.3389/fragi.2026.1724246

**Published:** 2026-07-15

**Authors:** Saumyadipta Pyne, Deep Ray, Meghana Ray

**Affiliations:** 1 Health Analytics Network, Columbia, MD, United States; 2 Department of Mathematics, University of Maryland, College Park, MD, United States; 3 HEED Lab, Columbia, MD, United States

**Keywords:** aging phenotype, bone mineral density, deep learning, generative adversarial network, osteoporosis, trajectory prediction

## Abstract

With a general increase in human lifespan, the need for technological advances to develop strategies for healthy aging has assumed great importance. In the present study, our goal is to predict the progression of selected aging phenotypes in a given healthy individual as they age past 65 years. Therefore, we developed a novel framework called dynamic views of aging with conditional generative adversarial networks (or DyViA-GAN) that can predict the plausible personalized trajectories of a selected aging phenotype conditioned on the available measurements of the phenotype at a few initial time instances and additional covariates. Given the prevalence of osteoporosis in the aging population, we selected the femoral neck bone mineral density (BMD) of a healthy individual as the phenotype of interest and baseline individual body mass index (BMI) as a covariate. We trained DyViA-GAN on a publicly available longitudinal dataset of a large cohort of mostly white women in the United States of age 65 years or above. It generated, for each individual, continuous phenotype trajectories, along with a corresponding region of acceptable predictions, for an age range of 66–89 years, for eight different combinations, both with and without involving the covariate. The prediction results were subjected to rigorous quality control and multiple comparative analyses. Our results clearly demonstrate the potential of generative deep learning frameworks in healthspan research.

## 1 Introduction

Forecasting the trajectories of diseases has been a cornerstone of the field of epidemiology, involving numerous approaches ranging from the classical susceptible-infectious-removed (SIR) and related models to more recent models based on time series and predictive analytics, genomics, etc. (Pyne et al., 2015). Modeling and prediction of infectious diseases have been among the key drivers of methodological advances in biostatistics and public health over the past century (Rao et al., 2017a; Rao et al., 2017b). Since the COVID-19 pandemic, novel approaches have emerged for outbreak forecasting, including artificial intelligence (AI)-driven deep learning of spatio-temporal patterns, prediction by real and synthetic data fusion, such as Zhang X. et al. (2021) and Fox et al. (2021). However, with a general increase in human lifespan over the past decades, some researchers in the field of disease prediction have shifted their attention from the modeling of infectious disease outbreaks in a population to modeling chronic conditions and their progression in individuals in the form of personalized trajectories. This work has led to such advances as deep aging clocks and various means of biological age assessment, such as Tian et al. (2023), Ecker et al. (2024), Jung et al. (2024), Shah et al. (2023), Raghu et al. (2021), and Putin et al. (2016).

In the present study, our goal is to predict the progression of selected aging phenotypes in a given healthy individual as they age past 65 years. Initial measurements of such phenotypes may be taken annually. With data from these initial time points as input, in combination with other static or dynamic risk factors as covariates, we aim to build a reliable generative AI model that is capable of forecasting future values of the same phenotypes for a particular individual. Thus, our model will produce a set of plausible trajectories (univariate or multivariate, depending on the selection of phenotypes) that could provide timely insights to inform and aid clinical decision-making that might extend a person’s *healthspan*. For example, trajectories of aging phenotypes associated with one’s cognition or bone health can help one strategically build preparedness or adopt preventive measures against future onset of dementia or possible fractures.

In particular, we focus on osteoporosis, which accounts for approximately 1.5 million fractures in the United States each year (Cooper et al., 1992). According to the National Institute of Aging (NIA)^1^, osteoporosis is a “silent disease” because it does not have any markers of progression until a bone breaks, usually in the hip, spine, or wrist. In severe osteoporosis cases, a simple movement such as a cough or minor bump can result in a broken bone, that is, a fracture. Additionally, individuals with osteoporosis may also have a longer duration of recovery and may experience chronic pain. In older adults, hip and spine fractures, in particular, can have serious consequences such as loss of mobility and independence. In a single typical year (2019), there were 318,797 emergency department visits, 290,130 hospitalizations, and 7,731 deaths related to hip fractures alone among adults 65 years or older in the United States (Moreland et al., 2023). Thus, osteoporosis acts as a key determinant of (bone) healthspan over the course of aging.

Osteoporosis is defined as a systemic skeletal disease that has both of the following characteristics: low bone mass and microarchitectural deterioration of bone tissues (NIH consensus development panel on osteoporosis prevention, diagnosis, and therapy, 2001). In clinical practice, osteoporosis is generally diagnosed when the measure of bone mineral density (BMD) of an individual is below the mean BMD of a reference population by at least 2.5 times the standard deviation, that is, a T-score ≤−2.5 (Kanis, 1994). Low BMD or osteopenia is determined by a T-score between −1 and −2.5 . As individuals age, BMD decreases, and consequently, osteoporosis becomes more prevalent in older adults (Hanley and Josse, 1996; Reid, 2010; Li et al., 2017). A study of BMD of the femoral neck or lumbar spine estimated that 43.4 and 10.2 million U.S. adults 50 years and above had low bone mass and osteoporosis, respectively (Wright et al., 2014). While several factors may be involved in the etiology of osteoporosis, historically, the body mass index (BMI) has been linked to bone health as a protective factor (Barrera et al., 2004; Reid, 2010). Using data from 8,600 postmenopausal women, Paganini-Hill et al. (1991) reported that high BMI was associated with a significant reduction in hip fracture risk independently of other potential confounders. Their findings were later corroborated by other studies (Cummings et al., 1995; DiPietro et al., 1993).

The longest-running cohort study in the United States on this topic is the National Institute of Aging (NIA)-sponsored Study of Osteoporotic Fractures (SOFs), a multi-site study that ran from 1986 to 2017 and recruited over 10,000 participants at four different sites. Initially, the study enrolled white women, and from 1997, it began to recruit African American women. Exclusion criteria included bilateral hip prostheses or the inability to walk without assistance. Study participants attended a series of clinical visits with physical and mental evaluations, including imaging to measure bone density, physical examinations, and questionnaires. In addition to clinical visits, participants were contacted via telephone or mail every 4 months to complete follow-up assessments for information on any falls, fractures, and vital status (SOF Online, 2020).

In a seminal article in 1995, the SOF researchers established the dual energy X-ray absorptiometry (DXA) scan as a non-invasive, painless, safe, and accurate measurement of BMD (Cummings et al., 1995). Subsequently, the U.S. Preventive Services Task Force osteoporosis management guidelines recommended routine BMD screening DXA scans for women 65 years of age or older (Nicholson et al., 2025; Kahwati et al., 2025). The SOF study also showed that women who fell and broke their hip were five times more likely to die in their first year of fracture than those who did not break their hip (SOF Online, 2020). Notably, beyond screening, DXA scans can also serve to monitor osteoporosis progression, leading to opportunities for intervention and prevention of fractures. Therefore, femoral neck BMD data, as measured by DXA, are an ideal initial choice of an aging phenotype to build a model for automated generation of personalized trajectories using a suitable computational framework.

Deep learning frameworks, such as long short-term memory networks (LSTMs), gated recurrent units (GRUs), and transformer networks, have been successful in applications involving time series forecasting (Giuliari et al., 2021; Siami-Namini et al., 2018; Zhang et al., 2024). The training and forecasting accuracy of these models typically rely on access to training data recorded over many time points. Many of the previous studies that applied deep learning for producing disease trajectories used one or more recurrent neural networks (RNNs) for predicting the ICD code for an individual’s next visit to the clinic (Choi et al., 2016a; Choi et al., 2016b; Rodrigues-Jr et al., 2021). Some have used generative adversarial networks (GANs) to predict such codes for multiple subsequent visits but not for tracking any actual phenotype of an individual (Shankar et al., 2023). Other deep learning applications have focused on specific health conditions or diseases, such as cystic fibrosis (Lim and van der Schaar, 2018) and Alzheimer’s disease (Fisher et al., 2019). Measures of DNA methylation biomarkers that estimate epigenetic aging serve a somewhat distinct purpose (Horvath and Raj, 2018; Belsky et al., 2022). Still other platforms, such as the Danish Disease Trajectory Browser (DTB), use statistical analysis of population-scale medical data to produce disease trajectories, but these are not personalized predictions (Siggaard et al., 2020). Various applications of generative AI and deep neural networks in aging research are described in reviews (Wilczok, 2025; Zhavoronkov et al., 2019) and the references therein.

To address such issues, we work with (a) individual longitudinal measurements of the selected phenotype that are available, and (b) only at a few (typically, 3–4) time points. The aforementioned deep learning models are not appropriate for predicting phenotype trajectories under such conditions. We instead appeal to deep generative models that are suitable for solving probabilistic problems. Among existing generative frameworks, conditional generative adversarial networks (cGANs) are popular in learning the underlying probability distribution of data conditioned on known values of other associated variables or parameters (Mirza and Osindero, 2014). cGANs have been successfully used in studies of medical images, including modeling the dynamics of cardiac aging (Campello et al., 2022) and Alzheimer’s disease progression based on MRI scans (Jung et al., 2023).

In this study, we proposed a novel cGAN framework called dynamic views of aging with GAN (or DyViA-GAN), which is capable of predicting the plausible personalized trajectories of a selected aging phenotype conditioned on the available measurements of the phenotype at a few initial time instances and additional covariates. For this purpose, we selected the BMD of the femoral neck of a healthy individual as the phenotype of interest and the baseline individual BMI as the covariate. Thus, DyViA-GAN generated personalized trajectories for eight different combinations, both with and without involving the covariate. The publicly available dataset used for training our model from the SOF study is described in Subsection 2.1 (Dataset). Then, we provide the details of the cGAN framework, including the network architectures and its model nomenclature for different data and covariate combinations in Section 2 (Methods). The predictions generated by DyViA-GAN were subjected to rigorous quality control and multiple comparative analyses. The results illustrate the corresponding cGAN-based generation of personalized trajectories of the selected phenotype. We conclude with Section 4, Discussion, which reviews some of the limitations of our study and the scope for future work.

## 2 Methods

### 2.1 Dataset

We worked with an anonymized dataset for 9,704 mostly Caucasian women participants in the now-concluded Study of Osteoporotic Fractures (SOF). The dataset is publicly available from the SOF Online website (https://sofonline.ucsf.edu/). Attendance during the study varied, as not all participants were available for all the visits, leading to significant gaps in the dataset. To maximize the number of participants in the training set, we considered participant data from Visit 2 (1989–90), Visit 4 (1992–94), Visit 5 (1995–96), and Visit 8 (2002–04), and considered the femoral neck BMD (denoted by FND) as measured by DXA scans using Hologic QDR 1000 workstations. In accordance with best practices, we exclude observations for patients over the age of 90 years to preserve patient anonymity (Moffatt and Leshin, 2024). We used longitudinally-adjusted scan measurements obtained by re-analyzing the previous scans and adjusting the regions-of-interest or deleting bone segments to match later scans. The T-scores of BMD were based on the parameters of a healthy reference population recommended by the International Society for Clinical Densitometry (ISCD) (Looker et al., 1997; Watts et al., 2013).

In total, we have 2,113 de-identified participant records, which were stratified based on the individual BMI values measured during Visit 1 (1986–87). We considered three strata: underweight and healthy (UWH) participants with BMI<25 , overweight (OW) participants with 25≤BMI<30 , and obese (OB) participants with BMI≥30 . Details of the samples and stratification can be found in Table 1. We also used the recorded age of the various participants during each of the four visits, with the ages rounded down to half years.

**TABLE 1:** Description of data (FND T-scores) used for training and testing. Stratification based on underweight and healthy participants (UWH) with BMI <25 , overweight participants (OW) with 25≤ BMI <30 , and obese participants (OB) with BMI ≥30 .

Data type	Strata	Number of participants	Min. FND	Max. FND	Mean FND ( ± SD)
Training	UWH	800	−4.63	4.94	-2.02 ( ± 0.87)
	OW	720	−5.07	2.51	-1.69 ( ± 0.87)
	OB	360	−3.97	1.70	-1.35 ( ± 0.92)
Test	UWH	98	−4.28	1.58	-2.04 ( ± 0.94)
	OW	95	−4.03	5.42	-1.70 ( ± 1.10)
	OB	40	−3.09	1.42	-1.44 ( ± 0.96)

### 2.2 DyViA-GAN

The proposed DyViA-GAN to predict personalized plausible trajectories of the selected phenotype comprises two neural networks, namely, the generator G and the discriminator D . We describe the algorithm for the most general setting, where the phenotype of interest is denoted by ρ and additional known covariates are denoted by Q . Here, ρ and Q may be a combination of different data modalities, such as anthropometry, images, lifestyle, cognitive functions, physical performance, and biomarkers. We assume access to a dataset from which we can extract the measured values of the desired phenotype(s) ρ at two sets of time points, ${\left\{t_{i}\right\}}_{i\in I}$ and ${\left\{t_{j}\right\}}_{j\in J}$ , where I and J are two disjoint (non-overlapping) time indexing sets. The corresponding phenotype values are denoted by ${\left\{\rho_{i}\right\}}_{i\in I}$ and ${\left\{\rho_{j}\right\}}_{j\in J}$ , respectively. Furthermore, the time-point sets ${\left\{t_{i}\right\}}_{i\in I}$ and ${\left\{t_{j}\right\}}_{j\in J}$ may be different and be unstructured across various records in the dataset.

The generator G is fed as input the vector $Y=\left(Q,{\left\{\rho_{i}\right\}}_{i\in I},{\left\{t_{i}\right\}}_{i\in I}\right)$ , a new time point t , and a random latent variable Z , which is sampled from an uncorrelated standard Gaussian distribution of dimension $N_{z}$ . The output of the generator is a predicted value of the phenotype in question at the time point t .

We use the notations $X={\left\{\rho_{j}\right\}}_{j\in J}$ and $T_{X}={\left\{t_{j}\right\}}_{j\in J}$ to denote the “true” paired data associated with a given vector Y . The corresponding predictions by the generator are denoted by $\hat{X}={\left\{{\hat{\rho }}_{j}\right\}}_{j\in J}$ , where ${\hat{\rho }}_{j}$ is the prediction at time point $t_{j}$ . The scalar-valued discriminator network D is fed as input either a “true” data tuple $\left(X,Y,T_{X}\right)$ or a “fake” tuple $\left(\hat{X},Y,T_{X}\right)$ . The role of the D is to distinguish between true and fake tuples, while the role of the G is to generate samples indistinguishable from those drawn from the true (unknown) conditional probability distribution of X given $\left(Y,T_{X}\right)$ , that is, $P_{X|\left(Y,T_{X}\right)}$ .

The two networks are trained in an adversarial fashion by solving the following optimization problem associated with a least-squares GAN (Mao et al., 2017):

$$\begin{array}{cc} D^{*} & =\arg\min_{D}\;\underset{P_{X,Y,T_{X}}}{E}\left[{\left(D\left(X,Y,T_{X}\right)-1\right)}^{2}\right]+\underset{P_{\hat{X},Y,T_{X}}}{E}\left[{\left(D\left(\hat{X},Y,T_{X}\right)\right)}^{2}\right]\\ G^{*} & =\arg\min_{G}\;\underset{P_{\hat{X},Y,T_{X}}}{E}\left[{\left(D\left(\hat{X},Y,T_{X}\right)-1\right)}^{2}\right], \end{array}$$ (1)

where $P_{X,Y,T_{X}}$ is the joint probability distribution of the true data, while $P_{\hat{X},Y,T_{X}}$ is joint probability distribution for the same data but with $\hat{X}$ generated by G .

Once the DyViA-GAN is trained, the optimized generator $G^{*}$ is used to generate an ensemble of possible values of phenotype ρ at any point t , given Y for an individual. This is achieved by drawing K random samples ${\left\{Z^{\left(k\right)}\right\}}_{k=1}^{K}$ of the latent variable and evaluating $G^{*}\left(Y,t,Z^{\left(k\right)}\right)$ for 1≤k≤K . By allowing t to vary in the entire temporal window of interest, we can generate an ensemble of plausible continuous trajectories of phenotype for an individual. These trajectories can then be used to compute the point-wise empirical mean and variance of the predicted trajectories (given a Y ) for any t as

$${\bar{\rho }}^{*}\left(t\right)=\frac{1}{K}\overset{K}{\underset{k=1}{\sum }}G^{*}\left(Y,t,Z^{\left(k\right)}\right),\text{Var}\left[\rho^{*}\right]\left(t\right)=\frac{1}{K}\overset{K}{\underset{k=1}{\sum }}{\left(G^{*}\left(Y,t,Z^{\left(k\right)}\right)-{\bar{\rho }}^{*}\left(t\right)\right)}^{2}.$$ (2)

**FIGURE 1:**
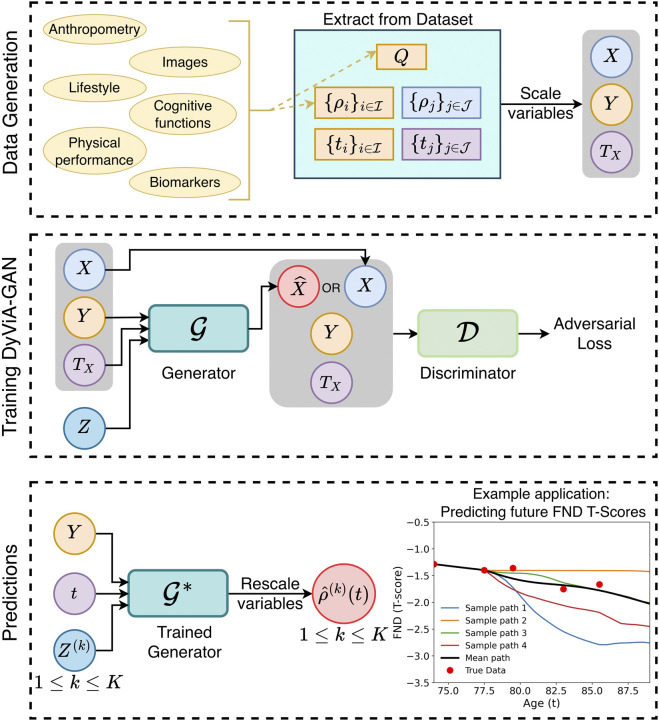
Schematic depicting the construction of the datasets, the inputs and outputs of G and D when training the DyViA-GAN, and how the trained generator $G^{*}$ is used to predict plausible phenotype trajectories. Top panel: The phenotype measurements, the time points of the measurements, and the additional covariates Q are extracted from the dataset to construct the tuple $\left(X,Y,T_{X}\right)$ after appropriate scaling. Middle panel: During the training phase, Y,Z and $T_{X}$ are fed to G to obtain the prediction vector $\hat{X}$ at the time points in $T_{X}$ . The discriminator D is fed either a true tuple $\left(X,Y,T_{X}\right)$ or a fake tuple $\left(\hat{X},Y,T_{X}\right)$ . Both networks are trained simultaneously according to Equation 1. Bottom panel: The trained generator $G^{*}$ is fed the conditional data Y for a participant, any time point t , and K samples of the latent variable. The output is an ensemble of K personalized trajectories ${\hat{\rho }}^{\left(k\right)}\left(t\right)$ for 1≤k≤K after rescaling the variables. As an example, we consider the FND prediction problem, illustrated with four sample trajectories and the mean trajectory (black curve), which can be compared with the known phenotype measurements (red dots).

A schematic of the training and evaluation of DyViA-GAN is shown in Figure 1.

For the study on the SOF dataset considered in the present work, ρ is taken to be the FND, while Q is chosen as the BMI measurement taken during Visit 1. Regression of the mean FND T-score against BMI in the data analyzed by this study shows a statistically significant positive association ( β=0.066 , ${\text{R}}^{2}=0.116$ , *p*-value = 0.017). Historically, it was shown that “A high body mass index protects against femoral neck osteoporosis in healthy elderly subjects” (Barrera et al., 2004). As noted in Subsection 2.1, we have FND values during visits 2, 3, 5, and 8. Thus, the index sets I and J used to construct $Y,X,T_{X}$ will be chosen as subset of {2,4,5,8} for training purposes. In particular, we set I={2,4} , while J={5} or J={5,8} . We scale the BMI by 100 before feeding it to the generator and scale the age of the participants in accordance with

$$t\to \frac{t-t_{\text{min}}}{t_{\text{max}}-t_{\text{min}}},\;t_{\text{min}}=66,\;t_{\text{max}}=89,$$ (3)

where $\left[t_{\text{min}},t_{\text{max}}\right]$ serves as the maximum age range (in years). The ages limits in (Equation 3) were chosen based on the data available in the SOF dataset. The above scaling (Rao et al., 2017b) ensures that t∈[0,1] when fed to the network as input. We also remark here that the ages $\left\{t_{2},t_{4},t_{5},t_{8}\right\}$ differ across participants in the dataset, thus adding a layer of complexity to the trajectory prediction problem.

### 2.3 Network architectures

Let |I| and |J| denote the size of the index sets. For the choices made in the present work, |I|=2 , while |J|=1 or 2. The generator G is taken to be a fully connected network with input dimension $2|I|+N_{z}+1$ (or $2|I|+N_{z}+2$ if BMI is used as a covariate) with a scalar-valued output. We used four hidden layers of width 50 each with the hat activation function (Hong et al., 2022; Zhang L. et al., 2021). To ensure that the generator’s prediction is constrained to pass through the given initial measurements corresponding to I , that is, at $t_{2}$ and $t_{4}$ , the output of the generator is transformed as follows:

$$G\left(Y,t,Z\right)\to \left[\left.G\left(Y,t,Z\right)\times \left(t_{4}-t\right)+{\text{FND}}_{4}\right)\right]\left(1-\phi \left(t;\sigma \right)\right)+\left[m\times \left(t-t_{4}\right)+{\text{FND}}_{4}\right]\phi \left(t;\sigma \right),$$ (4)

where

$$m=\frac{{\text{FND}}_{4}-{\text{FND}}_{2}}{t_{4}-t_{2}},\;\phi \left(t;\sigma \right)=\frac{1}{1+\exp\left(\left(t-t_{4}\right)/\sigma \right)}.$$

The function ϕ(t;σ) in the above transformation smoothly blends the linear curve passing through $\left(t_{2},{\text{FND}}_{2}\right)$ and $\left(t_{4},{\text{FND}}_{4}\right)$ to the network output. In this work, we choose $\sigma =10^{-2}$ .

The discriminator D is also taken to be a fully connected network with input dimension 2|I|+2|J| (or 2|I|+2|J|+1 if BMI is used as a covariate) with a scalar-valued output. We used four hidden layers of width 50 with the hat activation function. Other hyperparameter values for DyViA-GAN are listed in Table 2.

**TABLE 2:** Common hyperparameter values used for the DyViA-GAN models.

Hyperparameter	Value/choice
Latent dimension $N_{z}$	20
Optimizer	AdamW
Learning rate for G	$10^{-3}$
Learning rate for D	$10^{-3}$
Number of D updates per G update	4
$\ell^{2}$ regularization parameter	$10^{-7}$
Batch size	ALL: 200, OWH: 200, OW: 180, OB 90
Total epochs	2000

### 2.4 DyViA-GAN model nomenclature

We trained multiple versions of DyViA-GAN on different data and covariate combinations. For easier reference, we use the nomenclature Model- ⟨DT⟩ for the models trained on different data types determined by tag ⟨DT⟩ . The tag value ⟨DT⟩ = ALL implies that the model is trained on unstratified training data with 1,880 samples (see Table 1), ⟨DT⟩ = UWH implies the networks are trained on the UWH stratum samples with 900 samples, ⟨DT⟩ = OW implies training on the OW stratum with 800 training samples, while ⟨DT⟩ = OB implies training on the OB stratum with 400 training samples. The model name ends with an additional tag -Q if the covariate Q is fed as input to the generator. For example, Model-OWH denotes the model trained (and tested) on the OWH stratum, while Model-OB-Q denotes the model trained (and tested) on the OB stratum with Q used as an input for the generator. In summary, we consider eight DyViA-GAN models: 1) Model-ALL, 2) Model-ALL-Q, 3) Model-UWH, 4) Model-UWH-Q, 5) Model-OW, 6) Model-OW-Q, 7) Model-OB, and 8) Model-OB-Q. Furthermore, we consider two variants of the model, one with J={5} and another with J={5,8} . Thus, there are eight DyViA-GAN models for each variant.

### 2.5 Evaluation of model performance

Using the trained generator $G^{*}$ , we quantify the performance of the various models via the following metrics:

- Root mean squared error (RMSE): This will be evaluated either across all time points not fed as input to the generator (see Table 3) or at specific time points (see Table 4).
- Maximum score: For a particular participant, we define the score S(t) at any time point t using the empirical measures computed in Equation 2

$$S\left(t\right)=\frac{\left|\rho \left(t\right)-{\bar{\rho }}^{*}\left(t\right)\right|}{\sqrt{\text{Var}\left[\rho^{*}\right]\left(t\right)}},$$ (5)

where (Equation 5) represents is the normalized distance of the true value of the phenotype at t from the mean predicted value at that time point. The maximum score $S_{M}$ is defined as the maximum over an individual’s score over all time points not shown to the generator and at which point the true value is known. In the present context, $S_{M}=\max_{t\in \left\{t_{5},t_{8}\right\}}S\left(t\right)$ , with the empirical statistics computed using K=1000 random samples of Z . Thus, predictions with lower maximum scores are preferable. We say that the *score is acceptable* for a sample if $S_{M}\leq \epsilon_{s}$ where $\epsilon_{s}$ is a tunable parameter.

- Filtering plausible trajectories: to eliminate outliers from the predicted plausible trajectories for an individual, we perform an additional post-processing filtering. For each predicted plausible trajectory ${\hat{\rho }}_{k},\;1\leq k\leq K$ , consider the mean path $\bar{\bar{\rho }}\left[k\right]$ computed using the remaining K−1 trajectories, that is, excluding the k th trajectory. We then retain the k th trajectory if $\text{dist}\left({\hat{\rho }}_{k},\bar{\overset{¯}{\rho }}\left[k\right]\right)<\epsilon_{f}$ , where dist() is a meaningful function to measure the distance between trajectories, while $\epsilon_{f}$ is a tunable parameter. In the present work, we take dist() to be the dynamic time warping (DTW) distance, which is a commonly used elastic shape-based measure of similarity between a pair of time series (Dove et al., 2023).

**TABLE 3:** Percentage (%) of samples with acceptable scores $\left(S_{M}\leq 2.5\right)$ for 8 different DyViA-GAN models using J={5} (top sub-table) and J={5,8} bottom sub-table). The percentages are also listed for each data strata for models trained on unstratified samples. For models trained on stratified data, the percentages are listed only for the corresponding strata. The notation “-” indicates no scores are evaluated for a particular strata with a given model.

Models using J={5}								
	Training				Test			
Model Strata	UWH	OW	OB	ALL	UWH	OW	OB	ALL
Model-ALL	89.12	86.81	88.61	88.14	87.76	83.16	90.00	86.27
Model-ALL-Q	89.75	86.94	88.61	88.46	87.76	84.21	90.00	86.70
Model-UWH	85.00	-	-	-	77.55	-	-	-
Model-UWH-Q	91.62	-	-	-	87.76	-	-	-
Model-OW	-	84.72	-	-	-	82.11	-	-
Model-OW-Q	-	90.56	-	-	-	90.53	-	-
Model-OB	-	-	79.17	-	-	-	80.00	-
Model-OB-Q	-	-	84.17	-	-	-	85.00	-

Models using J={5,8}								
	Training				Test			
Model Strata	UWH	OW	OB	ALL	UWH	OW	OB	ALL
Model-ALL	82.62	78.33	81.11	80.69	82.65	80.00	87.50	82.40
Model-ALL-Q	80.00	76.11	79.44	78.40	79.59	78.95	87.50	80.69
Model-UWH	79.88	-	-	-	77.55	-	-	-
Model-UWH-Q	81.50	-	-	-	79.59	-	-	-
Model-OW	-	72.78	-	-	-	72.63	-	-
Model-OW-Q	-	90.56	-	-	-	90.53	-	-
Model-OB	-	-	72.22	-	-	-	77.50	-
Model-OB-Q	-	-	73.33	-	-	-	80.00	-

**TABLE 4:** RMSE for 8 different DyViA-GAN models using J={5} (top sub-table) and J={5,8} bottom sub-table). The errors are also listed for each data strata for models trained on unstratified samples. For models trained on stratified data, the errors are listed only for the corresponding strata. The notation “-” indicates no errors are evaluated for a particular strata with a given model.

Models using J={5}								
	Training				Test			
Model Strata	UWH	OW	OB	ALL	UWH	OW	OB	ALL
Model-ALL	0.53	0.57	0.52	0.54	0.56	0.68	0.57	0.61
Model-ALL-Q	0.55	0.59	0.53	0.56	0.59	0.69	0.58	0.63
Model-UWH	0.52	-	-	-	0.56	-	-	-
Model-UWH-Q	0.55	-	-	-	0.59	-	-	-
Model-OW	-	0.55	-	-	-	0.65	-	-
Model-OW-Q	-	0.55	-	-	-	0.66	-	-
Model-OB	-	-	0.59	-	-	-	0.64	-
Model-OB-Q	-	-	0.63	-	-	-	0.68	-

Models using J={5,8}								
	Training				Test			
Model Strata	UWH	OW	OB	ALL	UWH	OW	OB	ALL
Model-ALL	0.44	0.50	0.47	0.47	0.48	0.61	0.51	0.54
Model-ALL-Q	0.38	0.44	0.43	0.41	0.43	0.56	0.46	0.49
Model-UWH	0.38	-	-	-	0.43	-	-	-
Model-UWH-Q	0.39	-	-	-	0.44	-	-	-
Model-OW	-	0.45	-	-	-	0.56	-	-
Model-OW-Q	-	0.51	-	-	-	0.60	-	-
Model-OB	-	-	0.46	-	-	-	0.50	-
Model-OB-Q	-	-	0.44	-	-	-	0.48	-

We remark that the parameters $\epsilon_{s}$ used to define acceptable scores and $\epsilon_{f}$ used to filter trajectories are both problem-dependent and tunable by the practitioner based on domain expertise. In the present work, we choose $\epsilon_{s}=2.5$ and $\epsilon_{f}=2.0$ , determined by the ablation study presented in the Supplementary Material.

## 3 Results

We ran DyViA-GAN on the individual samples of available (or “true”) FND measurements from the dataset to generate their respective personalized continuous FND trajectories based on the stated models.

Using the criteria discussed in Subsection 2.5, we evaluate and compare the performance of the various designed models. Although Model-ALL and Model-ALL-Q are trained on unstratified data, we also evaluate the trained generators on stratified data for a more fine-grained analysis. The RMSE and maximum scores of the other models trained on stratified data are evaluated only within their respective strata.

### 3.1 Score-based comparison

When comparing the two variants of the model, Table 3 shows that a larger percentage of training and test samples have acceptable scores with models trained using J={5} than models trained using J={5,8} . This indicates that the plausible trajectories generated using the first variant have a larger spread around the predicted mean trajectory, thus having a better chance of capturing dynamics with larger temporal variations. We also observe that feeding the covariate Q (i.e., the BMI) as a generator input improves the performance of the models (both variants) trained on stratified data. For instance, the percentage of test samples with acceptable scores with models trained on the OW stratum increases from 82.11% to 90.53% for the first variant of the model (see the top sub-table in Table 3) and from 72.63% to 90.53% for the second variant of the model (see the bottom sub-table in Table 3).

### 3.2 RMSE-based comparison

If we consider the mean predicted trajectory as a single representation of the phenotype evolution, then the RMSE is a more appropriate metric to measure model performance. Table 4 shows that the models trained with J={5} (top sub-table) consistently lead to higher RMSE than the respective models trained with J={5,8} (bottom sub-table). Thus, based on the RMSE metric, the latter variant of the DyViA-GAN models performs significantly better. In general, the inclusion of the BMI as a generator input appears to marginally deteriorate the RMSE, except for the J={5,8} variant of Model-ALL-Q, in which the error reduces when BMI is included.

### 3.3 Checking and filtering predicted trajectories

**FIGURE 2:**
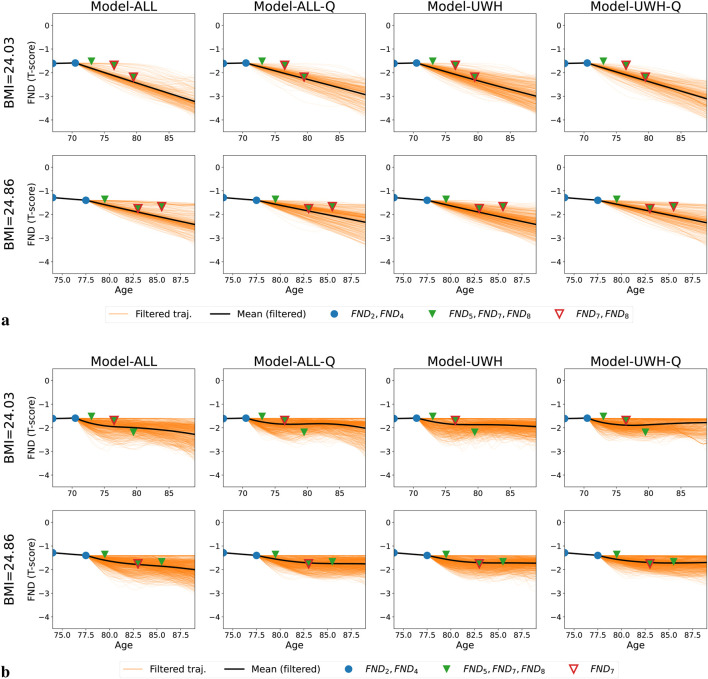
Predictions with various models for two test participants from the UWH stratum. Each plot depicts the filtered trajectories (orange curves) out of 1,000 original trajectories, the mean filtered personalized phenotype trajectory (black curve), and the phenotype measurements at times $t_{2},t_{4},t_{5},t_{7},t_{8}$ (dots and triangles). We depict the generator input using blue dots, the generator predictions using green triangles, and mark the prediction at Visit 7 (never shown to the DyViA-GAN) using red (outlined) triangles. The participant’s BMI is listed on the left of each row. **(a)** Results for models using J={5} . **(b)** Results for models using J={5,8} .

**FIGURE 3:**
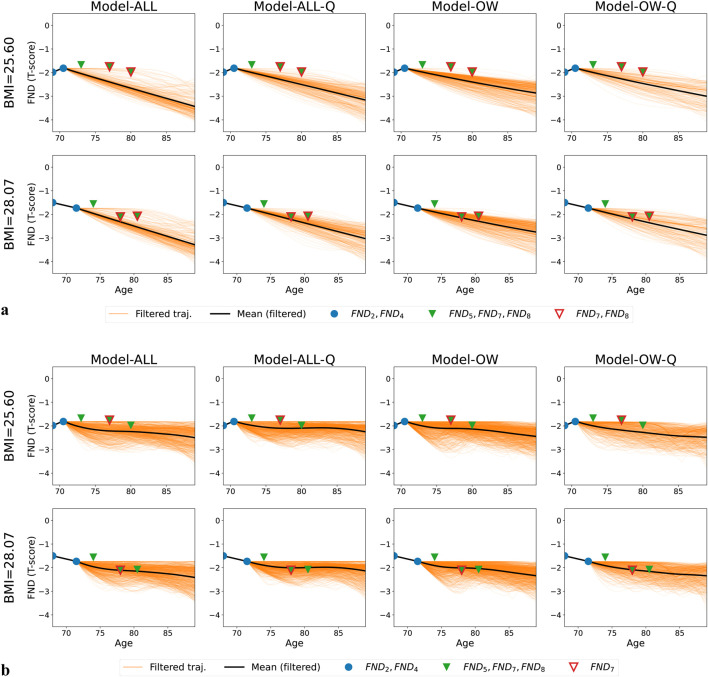
Predictions with various models for two test participants from the OW strata. Each plot depicts the filtered trajectories (orange curves) out of 1,000 original trajectories, the mean filtered personalized phenotype trajectory (black curve), and the phenotype measurements at times $t_{2},t_{4},t_{5},t_{7},t_{8}$ (dots and triangles). We depict the generator input using blue dots, the generator predictions using green triangles, and mark the prediction at Visit 7 (never shown to the DyViA-GAN) using red (outlined) triangles. The participant’s BMI is listed on the left of each row. **(a)** Results for models using J={5} . **(b)** Results for models using J={5,8} .

**FIGURE 4:**
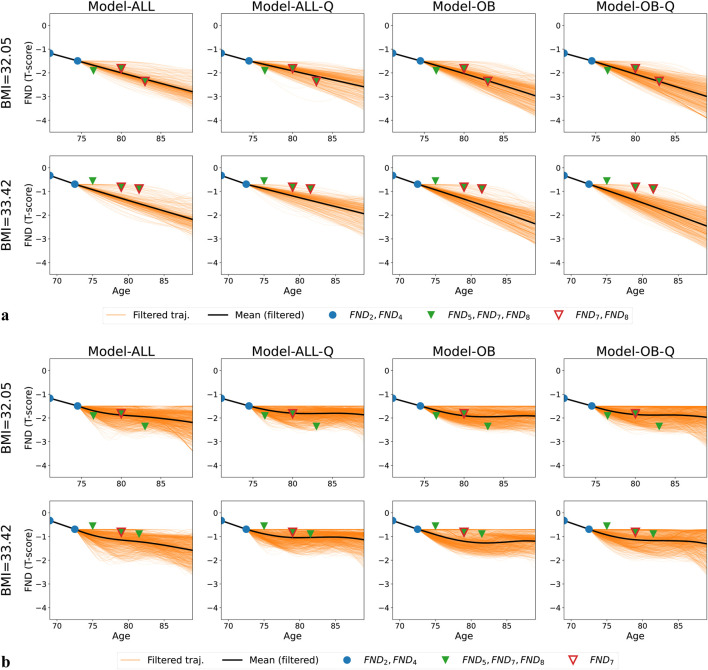
Predictions with various models for two test participants from the OB strata. Each plot depicts the filtered trajectories (orange curves) out of 1,000 original trajectories, the mean filtered personalized phenotype trajectory (black curve), and the phenotype measurements at times $t_{2},t_{4},t_{5},t_{7},t_{8}$ (dots and triangles). We depict the generator input using blue dots, the generator predictions using green triangles, and mark the prediction at Visit 7 (never shown to the DyViA-GAN) using red (outlined) triangles. The participant’s BMI is listed on the left of each row. **(a)** Results for models using J={5} . **(b)** Results for models using J={5,8} .

With the goal of eliminating outliers among the predicted trajectories, we filter the trajectories generated by the DyViA-GAN models using DTW with a filtering parameter $\epsilon_{f}=2.0$ . We demonstrate the qualitative performance of the trained models with this filtering on two test participants chosen from each stratum: UWH, OW, and OB. For each participant, we generate K=1000 trajectories using each model and then compute the personalized mean trajectory only using the filtered trajectories. The filtered trajectories and corresponding mean trajectories are shown in Figure 2 (OWH), Figure 3 (OW), and Figure 4 (OB).

In addition, we have access to the adjusted phenotype value at Visit 7 (1999) for these six test participants. This serves as held-out data points for both variants of DyViA-GAN, as data from this visit were not used during the training phase. Note that Visit 8 is also a held-out data point for the models trained with J={5} . We depict the generator inputs using blue dots, the generator predictions using green triangles, and mark the unseen/held-out time points using red (outlined) triangles in Figures 2–4. The Supplementary Material contains additional plots depicting a few sample trajectories for each of these test participants for better clarity. We make the following observations from the plots in Figures 2–4:

- The mean trajectories pass through the true data at $t_{2},t_{4}$ , which is a feature of the constraint Equation 4 applied on the generator output.
- With the filtering threshold $\epsilon_{f}=2.0$ , a significantly larger number of plausible trajectories are accepted with the J={5,8} model variants than the J={5} variants. This can be easily inferred by observing the density of the filtered trajectories shown in the figures.
- The filtered trajectories with the J={5,8} models are significantly more nonlinear with richer structure, while those obtained using the J={5} models appear to be mostly (piecewise) linear (also see Supplementary Material).
- The (filtered) mean with the J={5,8} models leads to a better approximation of the unseen data at future time point $t_{7}$ .
- The mean trajectory of all models generally predicts a downward trend in the FND values as time progresses, which is consistent with clinical observations in aging patients.

### 3.4 Benchmarking against other methods

We compare the proposed DyViA-GAN against other standard approaches. In particular, we consider a standard cubic smoothing spline model, linear mixed-effect models (LMMs), and Gaussian process (GP) regression. Among these, the latter two approaches are well suited to longitudinal data with unstructured time points.

#### 3.4.1 Cubic splines

We use a standard cubic smoothing spline model (Fritsch and Carlson, 1980) as implemented by the “splinefun” function in R. The advantage of such a model (say, over a full ARIMA model) is that it not only provides a smooth historical trend but also a linear forecasting ability, which is hardly affected by the restricted parameter space (Hyndman et al., 2005). The dataset to train the spline model is constructed by considering the maximum number of participants in the SOF dataset who have FND measurements from visits 2, 4, 5, and 8 while also sharing the same ages $t_{2},t_{4},t_{5}$ and $t_{8}$ during these visits. This yields 49 participants with (unscaled) ages of 69.0 years, 72.5 years, 74.5 years, and 81.5 years. The spline model was trained on the first two ages and then used to predict the T-scores during the last two ages. We compare the RMSE of the spline predictions with the mean predictions at these two ages using the various DyViA-GAN models trained previously on unstratified data, that is, Model-ALL and Model-ALL-Q. As shown in Table 5, the DyViA-GAN models outperform the spline model when predicting the T-scores at 74.5 years. However, only the J={5,8} variants lead to lower errors than splines at age 81.5 years. Furthermore, including BMI as input improves the performance of this latter variant, which is also consistent with the observations from Table 4.

**TABLE 5:** Comparing the performance of Model-ALL and Model-ALL-Q with a spline-based regression model. The RMSE of the predictions at ages 74.5 and 81.5 years are shown.

	J={5}		J={5,8}		Splines
Age Model	Model-ALL	Model-ALL-Q	Model-ALL	Model-ALL-Q	
74.5	0.26	0.26	0.27	0.26	0.32
81.5	0.70	0.72	0.57	0.51	0.67

#### 3.4.2 Linear mixed-effects models

We consider LMMs with fixed (population-level) effects modeled using a polynomial basis (in time) of dimension p and random (individual-level) effects modeled using a polynomial basis of dimension q . The random basis coefficients are modeled using a normal distribution N(0,Σ) , and additional (individual-level) observation noise is modeled using $N\left(0,\sigma^{2}I\right)$ . The coefficients of the fixed basis, covariance Σ for the random coefficients, and $\sigma^{2}$ for the noise model are determined by maximizing the log-likelihood on the (unstratified) training data. Once trained, the predictions for an individual are conditioned on their measurements at the first two time points. We use the nomenclature LMM (p,q) to denote the LMM trained with basis dimension p and q .

**FIGURE 5:**
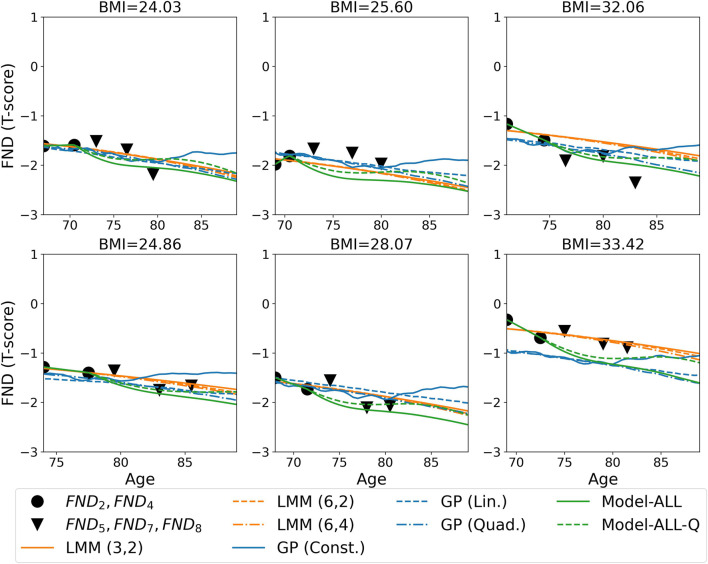
Comparing the mean predicted trajectories with LMMs, GPs, Model-ALL, and Model-ALL-Q, with J={5,8} for the latter two approaches. The six test participants are the same as those considered in Figures 2–4. Each plot depicts the phenotype measurements at the initial time points $t_{2},t_{4}$ (black dots) that are fed to the models as input and future time points $t_{5},t_{7},t_{8}$ (black triangles). The mean trajectories with the LMM models are shown in orange, the GPs are shown in blue, and the DyViA-GAN are shown in green. The participant’s BMI is listed at the top of each plot.

We consider three variants of LMM based on the values of p and q . As shown in Table 6, the RMSE (of 0.49) on the test samples remains fairly robust across all variants, which perform better than Model-ALL and at par with Model-ALL-Q. We also compare the performance of the models on the six test participants considered in the previous sections. Figure 5 shows that all LMMs predict fairly linear (mean) trajectories. However, this is not ideal when the individual-level evolution of phenotypes is non-monotonic, as shown in Figure 5 for participants with BMIs of 25.60 and 32.06. In comparison, the DyViA-GAN predictions are far more expressive and can capture non-monotone behavior. Furthermore, we have the flexibility of choosing a network architecture that ensures the predictions pass through (or close to) the given observations, which need not hold true for LMMs.

**TABLE 6:** RMSE of the predictions of various models on the unstratified test data.

Model	RMSE
LMM (3,2)	0.49
LMM (6,2)	0.49
LMM (6,4)	0.49
GP Const.	0.62
GP Lin.	0.61
GP Quad.	0.62
Model-ALL	0.54
Model-ALL-Q	0.49

#### 3.4.3 Gaussian process regression

We consider GP models with mixed effects. For individuals i and j whose FND values are observed at times t and $t^{\prime }$ , the mixed kernel is taken as $k\left(\left(i,t\right),\left(j,t^{\prime }\right)\right)=k_{\text{pop}}\left(t,t^{\prime }\right)+\delta_{i,j}k_{\text{indiv}}\left(t,t^{\prime }\right)$ , where $k_{\text{pop}}$ models the population-level dynamics, $k_{\text{indiv}}$ models the individual-level dynamics, and $\delta_{i,j}=1$ only if i=j (i.e., both measurements are for the same individual). In the present work, we take $k_{\text{pop}}$ to be the Matérn kernel with parameter ν=2.5 , and we take $k_{\text{indiv}}$ to be a rougher Matérn kernel with ν=0.5 . Furthermore, we consider the mean of GP to be either a constant mean, linear, or quadratic. The coefficients of the mean and kernel length-scales are determined by maximizing the log-likelihood on the (unstratified) training data. Similar to the LMMs and DyViA-GAN, the predictions with trained GP models are conditioned on the measurements at the first two time points. We use the nomenclature GP Const., GP Lin., and GP Quad., based on how the mean is modeled.

We observe from the RMSE comparison in Table 6 as well as the predicted mean trajectories in Figure 5 that the GP models perform poorly compared to LMMs and DyViA-GAN. Unlike the LMMs, the predictions with the GPs are not linear and appear to possess similar expressivity to DyViA-GAN. Thus, it may be possible to fine-tune the GP or choose a more sophisticated GP variant to improve its performance. This will be explored in future extensions of this work.

## 4 Discussion

While noting that the concept of *healthspan* is relatively new in geroscience research (Burch et al., 2014) and there is as yet no universal agreement on its definition, the author of a perspective article in *GeroScience* made two key observations: (i) “health itself may be better considered a continuous variable that changes in a dynamic way throughout life”, and (ii) “[t]he health trajectory will be different in different individuals but will generally trend downward with age” (Kaeberlein, 2018). Understanding and prolonging individual-specific healthspan is challenging for many reasons, including not being aware of the trajectories of the different aging phenotypes. Advance knowledge of the progression of such an outcome as osteopenia, the precursor of osteoporosis, is crucial as it can lead to an accelerated reduction in one’s bone healthspan, and DyViA-GAN provides precisely that capability.

While there are several powerful approaches that one could use for the trajectory prediction problem, we used a GAN-based approach motivated by the following considerations:

- GANs allow for the generation of many possible outputs associated with a given input. In other words, it is a probabilistic method versus a deterministic one.
- Deep learning frameworks, such as GANs, allow the easy integration of different data modalities. For example, we can easily handle input comprising CT scans (i.e., image-type data) and femoral neck density at different time points (i.e., longitudinal scalar data). Furthermore, we have the flexibility of having a different data type for the predictions. This level of flexibility is seldom granted by more traditional algorithms.
- Once trained, inference/predictions on new data are instantaneous. This is an advantage over more sophisticated generative algorithms such as diffusion models (Cao et al., 2024).

The present study has some distinct advantages. Using a (very) few initial observations as input, it demonstrated the capability of cGAN-type models to predict the personalized trajectory of a single phenotype, the femoral neck BMD, which is particularly relevant to a patient’s healthspan, and increasingly so with age. The generators of the proposed DyViA-GAN take as input the (scaled) age variable t , and the output generated is a *continuous* trajectory for the entire age span, not only for some discrete time points at which data are available during training/testing. The continuous representation also allows for easy calculation of the gradient (and higher derivatives) of each trajectory using automatic differentiation on neural networks.

Notably, there are several key findings from the results presented in Section 3. First, the score-based metric indicates that the J={5} DyViA-GAN models generate more spread-out trajectories. However, this might lead to the generation of outlier trajectories that can skew statistical predictions. This was observed when using the RMSE metric, which clearly showed that J={5,8} DyViA-GAN models lead to better predictions (in the mean), even when compared with a standard spline-based regression model. Second, the inclusion of additional risk factors, such as BMI, may lead to better personalized predictions in certain situations. Third, the J={5} models lead to the generation of simplistic trajectories compared to J={5,8} , indicating the latter are more suitable to capture nonlinear dynamics. Fourth, filtering the predicted trajectories provides a useful strategy to eliminate outliers, leading to improved predictions for the unseen data from Visit 7. Fifth, the Model-ALL-Q variant of DyViA-GAN is at par with standard LMMs in terms of the RMSE metric while being more expressive when predicting individual-level non-monotone trajectories. Moreover Model-ALL-Q and LMMs outperform the GP models.

According to the U.S. Census Bureau’s 2017 National Population Projections, the year 2030 will mark an important demographic turning point in U.S. history, when one in five Americans will be 65 years or older. Clearly, planning for and achieving individual healthspan goals will become increasingly important in society. Toward this, new sources of rich, high-resolution data, such as genomics, epigenetics, wearable monitoring devices, etc., are making healthcare more preventive and personalized. Such data also allow for complex interplays among the factors of healthy aging, ranging from static to dynamic, and both internal and external, to inform the models for predicting a wider and more insightful set of outcomes. For instance, a better understanding of the nonlinear relationship between one’s BMI and BMD, over the course of aging, could emerge (Cummings et al., 1995). Toward this end, the translational capacity of AI-based approaches to preventive and precision medicine, including the present platform, can be refined and tested in clinical trials.

We understand that there are certain limitations of our study. As future work, our approach could be extended to simultaneously predict the trajectories of multiple phenotypes, say, BMD values of multiple regions of the body or fusion thereof. Moreover, key insights are available from other markers of osteoporosis beyond BMI, which we chose for illustrative purposes and for its easy availability and relevance, such as Barrera et al. (2004). DyViA-GAN has the flexibility to include, in addition to static baseline risk factors, dynamic covariates such as a participant’s physical activity level, chronic inflammation, bone biomarkers, epigenetic changes, etc. The effect of including such covariates in the trajectory prediction problem will be considered in future extensions. We also plan to train the models with longitudinal BMD data from heterogeneous populations, such as He et al. (2023). The presently generated trajectories are not dynamically updated but could be revised in the future to allow for intermediate events, lifestyle alterations, and medical interventions. Further, we plan to extend the application to more sophisticated deep learning architectures to build the cGAN generator that accounts for the temporal nature of data (e.g., RNN and LSTM), alternative deep generative frameworks (Batzolis et al. (2021) and Dasgupta et al. (2025)), as well as other relevant longitudinal datasets on aging phenotypes and groups to train our generative models (Cummings et al. (2006)).

## Data Availability

The original contributions presented in the study are included in the article/Supplementary Material; further inquiries can be directed to the corresponding author.

## Footnotes

1
https://www.nia.nih.gov/health/osteoporosis/osteoporosis
