# Supplementary material for: Automated generation of personalized trajectories of aging phenotypes with DyViA-GAN
Source: Front Aging. 2026 Jul 15;7:1724246. doi: 10.3389/fragi.2026.1724246 (PMC13416555; doi:10.3389/fragi.2026.1724246)
Supplement: Supplementary file 1 [file Supplementaryfile1.pdf]

Supplementary Material

1 ADDITIONAL RESULTS

As a companion to the test samples considered in Figure 2,3 and 4 in the main manuscript, we plot a few (filtered) sample trajectories predicted by DyViA-GAN for each of these test samples. These are shown in Supplementary Figures Figure S1, Figure S2 and Figure S3 below.

2 ABLATION STUDY: CHOOSING ϵ_s AND ϵ_f

To determine a suitable value of ϵ_s which is used to define the acceptable scores, we study how the percentage of test samples with an acceptable score, i.e., $\mathcal{S}_M \leq \epsilon_s$, changes as ϵ_s is increased. Figure Figure S4 plots this change with Model-ALL and Model-ALL-Q (with $\mathcal{J} = \{5, 8\}$), both of which are trained on the same unstratified dataset. We observe a sharp transition near $\epsilon_s = 2.5$ at which point about 80% of the test sample predictions have acceptable scores. Beyond this threshold, there is a very gradual change in the influence of ϵ_s over the acceptable scores of test samples. Thus, we choose $\epsilon_s = 2.5$ in our experiments.

Next, we determine a suitable value for ϵ_f which is used to filter the samples trajectories on individuals based on DTW distance. In Figure Figure S5, we plot the fraction of trajectories predicted (by Model-ALL-Q) that are retained for various values of ϵ_f. The six colored solid dotted lines show the fractions for the same six test participants considered in the previous section, while the black dashed dotted line shows the median fractions for each value of ϵ_f considered. We observe a sharp transition near $\epsilon_f = 2.0$, at which roughly 88% of trajectories are retained. The fraction increase more gradually beyond this value of ϵ_f. This motivates our choice for $\epsilon_f = 2.0$ in our experiments.

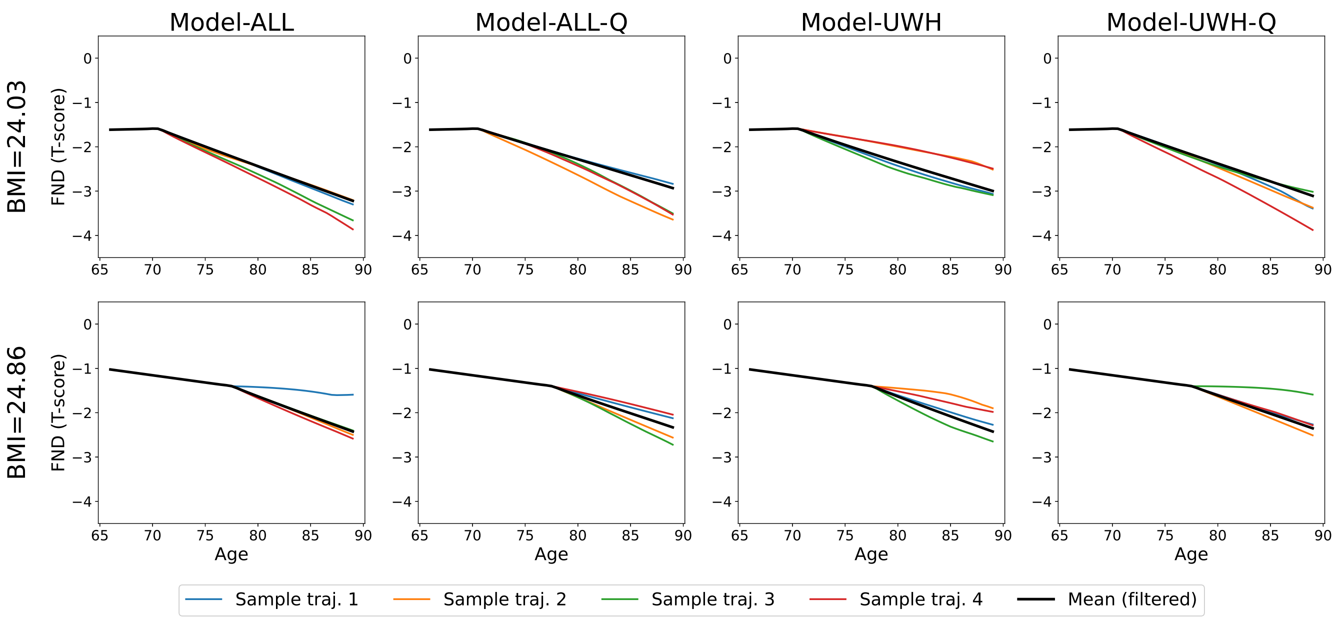

a

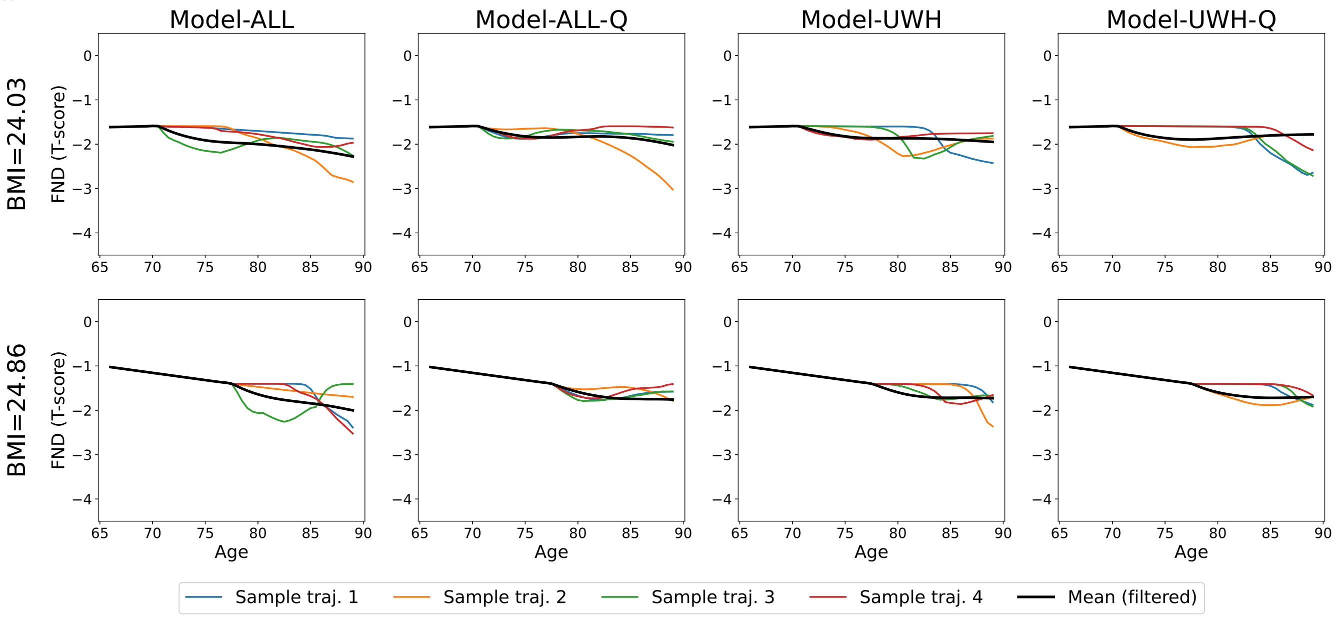

b

Figure S1. Predictions with models for two test participants from strata UWH. Each plot depicts the (filtered) mean personalized phenotype trajectory (black curve) and four (filtered) sample trajectories. The BMI of the participant is listed on the left of each row. **(a)** Models using $\mathcal{J} = \{5\}$. **(b)** Models using $\mathcal{J} = \{5, 8\}$.

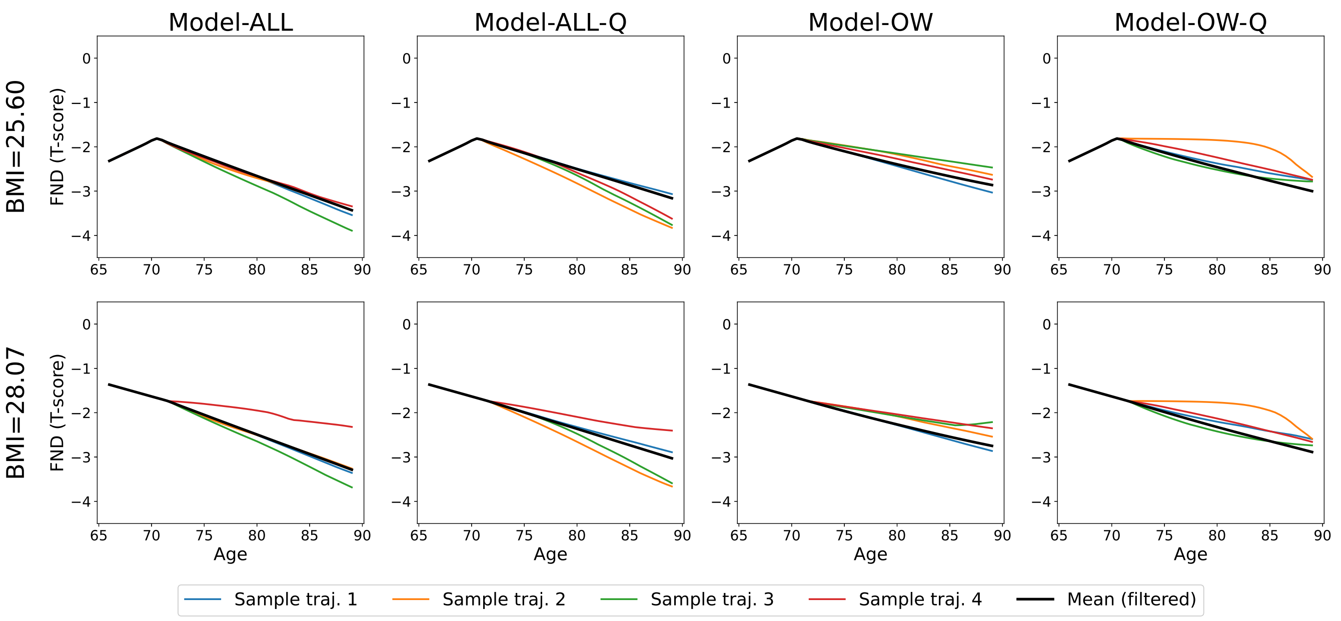

a

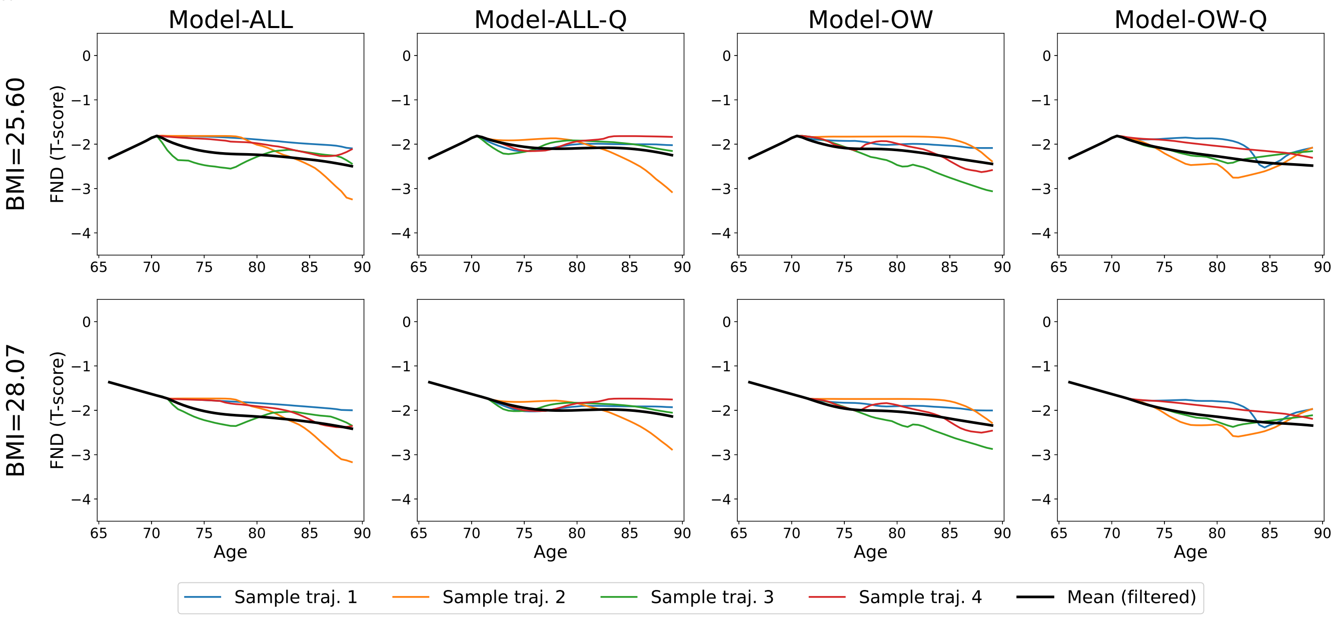

b

Figure S2. Predictions with models for two test participants from strata OW. Each plot depicts the (filtered) mean personalized phenotype trajectory (black curve) and four (filtered) sample trajectories. The BMI of the participant is listed on the left of each row. **(a)** Models using $\mathcal{J} = \{5\}$. **(b)** Models using $\mathcal{J} = \{5, 8\}$.

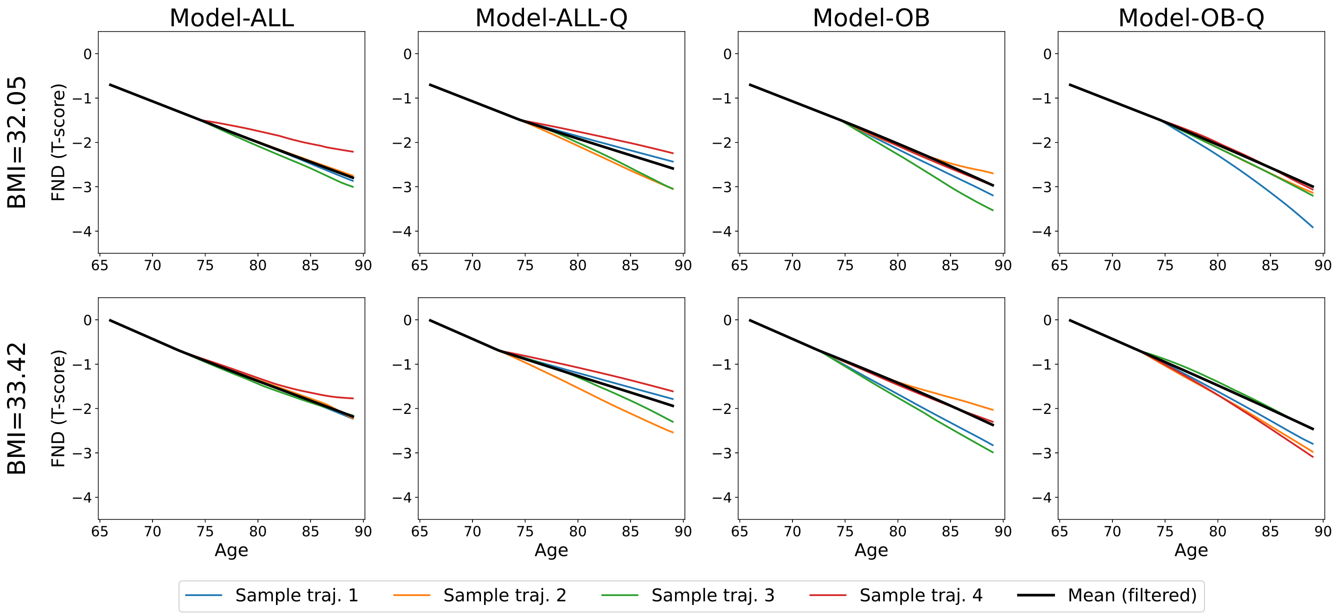

a

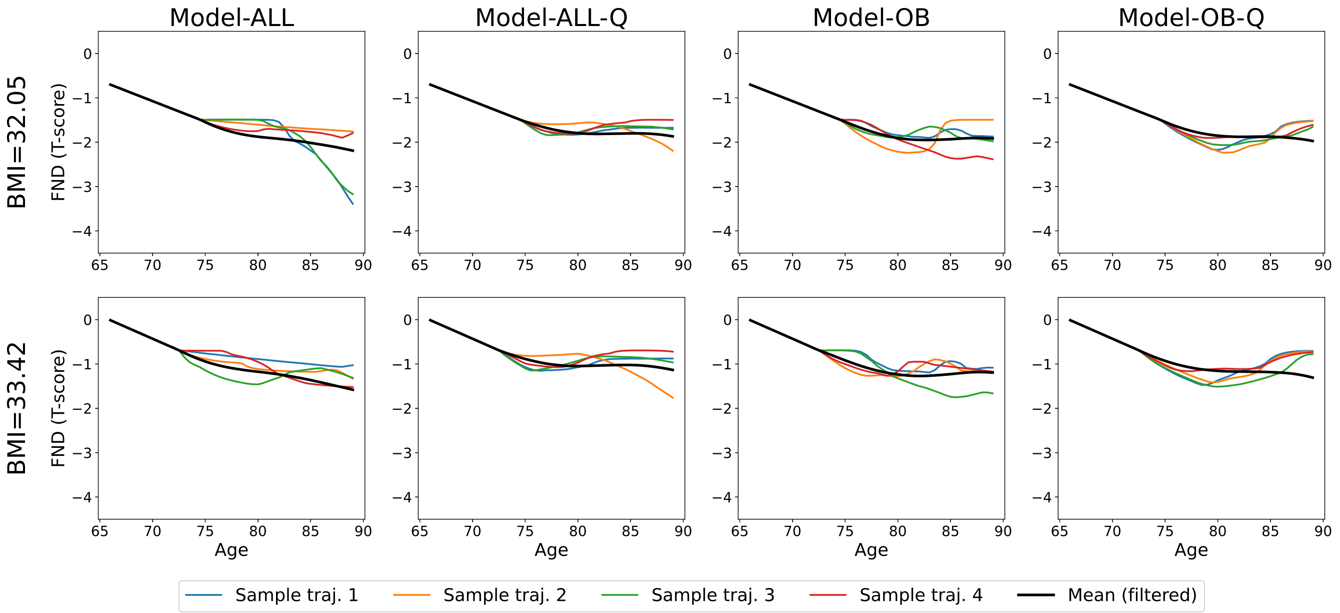

b

Figure S3. Predictions with models for two test participants from strata OB. Each plot depicts the (filtered) mean personalized phenotype trajectory (black curve) and four (filtered) sample trajectories. The BMI of the participant is listed on the left of each row. **(a)** Models using $\mathcal{J} = \{5\}$. **(b)** Models using $\mathcal{J} = \{5, 8\}$.

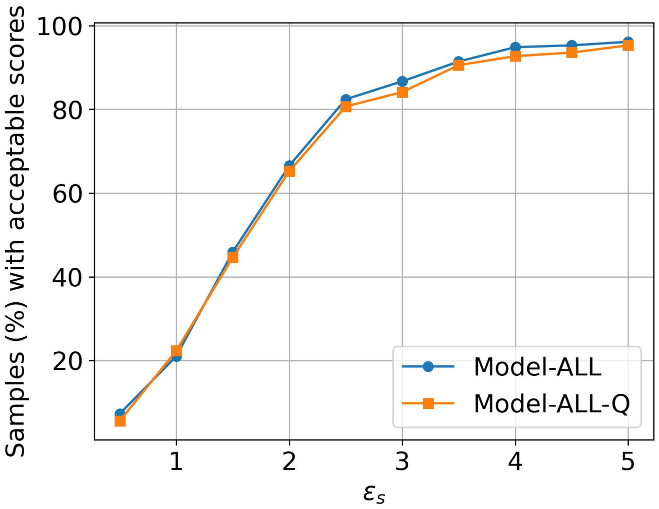

Figure S4. Ablation study to understand the effect of ϵ_s in selecting samples with an acceptable score. The results are shown for Model-ALL (blue curve) and Model-ALL-Q (orange curve), with the y-axis indicating the percentage of test samples flagged as have a suitable score, i.e., $\mathcal{S}_M \leq \epsilon_s$.

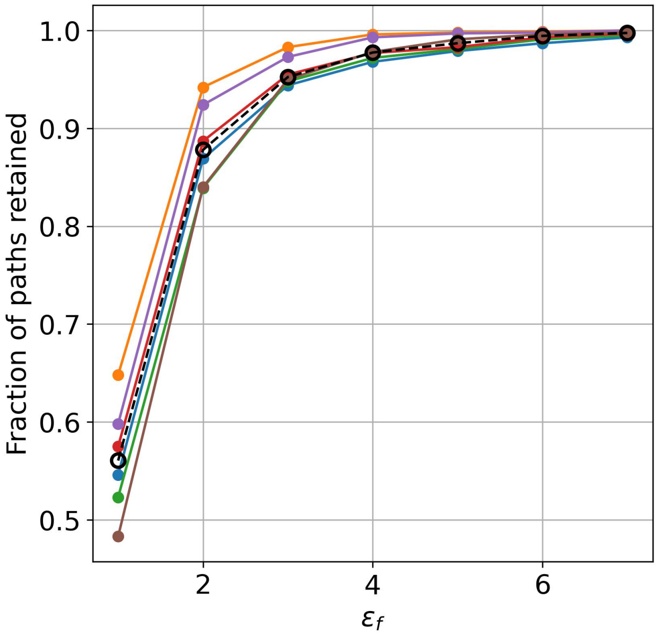

Figure S5. Ablation study to understand the effect of ϵ_f in filtering trajectories based on the DTW distance for different individuals. The colored solid dotted lines show the fraction of predicted trajectories retained as ϵ_f is varied for six test participants for illustrative purposes. The black dashed dotted line shows the median fractions for each value of ϵ_f considered.
